# Effects of Mother’s Dominance Hierarchy on the Development of Social Relationships among Immature Tibetan Macaques

**DOI:** 10.3390/ani12070904

**Published:** 2022-04-01

**Authors:** Chuan-Chang Liu, Shi-Wang Chen, Qi-Bing Wei, Bing-Hua Sun, Xi Wang, Dong-Po Xia

**Affiliations:** 1 School of Life Sciences, Anhui University, Hefei 230601, China; liuchuanchang1997@163.com (C.-C.L.); chenwang1962@163.com (S.-W.C.); dairysike@163.com (Q.-B.W.); 2 International Collaborative Research Center for Huangshan Biodiversity and Tibetan Macaque Behavioral Ecology, Hefei 230601, China; binghuasun00@126.com (B.-H.S.); wangxi198307@163.com (X.W.); 3 School of Resource and Environmental Engineering, Anhui University, Hefei 230601, China

**Keywords:** *Macaca thibetana*, mother’s rank, social play, social grooming

## Abstract

**Simple Summary:**

In this study, we explored the role of females’ social ranks on social behaviors among immature Tibetan macaques (*Macaca thibetana*). The results suggest that females’ social ranks affected their offspring’s social play and grooming during the juvenile and adolescent periods, but not the infancy period. The present study provides new insight into understanding the effects of the female dominance hierarchy on the development of social relationships among immature offspring in nonhuman primates.

**Abstract:**

During a relatively long period of growth, immature individuals rely on their mothers to obtain nutrition, and a good environment for learning social skills needed to cope with complex environments in adulthood. In this study, we collected the behavioral data of Tibetan macaques (*Macaca thibetana*) to investigate the effects of females’ social rank on the development of social relationships among their immature offspring from November to June 2021. The results show that there was no difference in the rate/type of social play and grooming among infants. However, among juveniles and adolescents, the higher their mother’s social rank, the higher the rate of social play they participated in, and the more aggressive play they engaged in. Immatures with high-ranking mothers initiated more social play among each other. A similar pattern of playmates was found among juveniles/adolescents with middle-ranking and low-ranking mothers. We also found that immatures preferred immatures with higher-ranking mothers as grooming mates and initiated more grooming with immatures with higher-ranking mothers than with those with lower-ranking mothers. Our study suggests that females’ social ranks affect the development of social relationships among their immature offspring. In despotic nonhuman primates, this indicates that the mother’s dominance hierarchy would directly or indirectly influence the processes of participating in social interactions and choosing partnerships among immature individuals with age (i.e., infancy, juvenile, and adolescent periods).

## 1. Introduction

In nonhuman primates, immature individuals experience a relatively long period of growth and development [1]. During this period, immatures are vulnerable and completely incapable of independent survival [2]. Thus, they must rely on their mothers to provide nutrition and a social environment to participate in social interactions for promoting individual development and form social relationships to cope with a complex society in adulthood [3,4].

Social play and social grooming have been documented as the main and typical social interactive behaviors during the immature periods in nonhuman primates [5]. The main function of social play serves to accelerate motor and physical skills and improve individual cognitive and social skills (the motor training hypothesis and social skill training hypothesis [6,7,8]). Previous studies have also stated that social play facilitates the development of communicative skills and forms the foundation for adult social relations [9]. Social play usually decreases with age and is rarely observed in adulthood, whereas the frequencies of affiliative interactions increase with age [10,11]. Social grooming, which is considered as a typical affiliative interaction, represents the extent of social relationships between individuals in nonhuman primates (such as rhesus macaques, *Macaca mulatta* [12]; bonnet macaques, *Macaca radiata* [13]; barbary macaques, *Macaca sylvanus* [14]). Accordingly, references to the functions of social play and social grooming have been focused on studying social relationships among immatures [15,16,17].

An increasing number of studies have shown that the effects of mothers’ social ranks on the social play and social grooming of their immature offspring were complex [18]. Firstly, the mother’s effects might vary among species. For example, some studies proposed that infants with high-ranking mothers participated in a higher rate of social play than those with low-ranking mothers, as found in rhesus macaques (*Macaca mulatta*) [19,20]. However, some researchers argued that the mother’s social rank did not influence the rate of social play among infants, as found in golden snub-nosed monkeys (*Rhinopithecus roxellana*) [21]. Moreover, even within the same species, the outcomes of the mother’s effects would vary according to studies conducted in different study sites. For example, in vervet monkeys (*Cercopitheeus aethiops*) living in East Africa, immatures with high-ranking mothers received more grooming than they gave [22]. However, no correlation was found between social grooming and the mother’s social rank in the same species living in South Africa [23]. In addition, the mother’s effects would also vary among different age-class immature individuals within a social group. For example, previous studies found that the amount of social play of yearling rhesus macaques was positively correlated with their mothers’ social rank, whereas a negative correlation was found between these two variables for juveniles [20].

Cross- and within-species comparison studies have provided an empirical framework to identify the social or ecological factors underlying similar and different outcomes between immatures’ social interactions and their mothers’ social rank. To facilitate the understanding of the effects of the mother’s dominance hierarchy, the dynamic of the relationship between social interactions and the mother’s social rank with age, rather than the outcomes, should be paid more attention. In primate species, most mothers start out more protective and restrictive toward their infants. Additionally, as the offspring grow up, they become more laissez-faire [24]. This indicates that the role of the mother changes with the age of the offspring, which could affect the social development of immature individuals. Accordingly, the effects of the mother’s dominance hierarchy would be dynamic processes of participating in social interactions and choosing partnerships among immature dyads with age (i.e., infancy, juvenile, and adolescent periods), especially in a despotic species. However, less is known about this process.

Tibetan macaques (*Macaca thibetana*) are an endemic nonhuman primate species found in China. They live in multi-male and multi-female social groups [18]. In a social group, more than half of the group members are immature individuals and live with their mothers [25]. Tibetan macaques are a female-philopatric and male-dispersed species [25]. Males and females attain sexual maturity by age seven and five, respectively [18]. Thierry [26] classified the Tibetan macaque as grade 3 with a tolerant social style. However, according to the low rates of counter-aggression and low conciliatory tendencies based on a field study, Berman et al. argued that the species should be reclassified as grade 2 with a despotic society [27]. Thus, Tibetan macaques form a strict linear dominance hierarchy [27]. The social ranks of immature individuals depend on the hierarchical status of their mother, where the higher the rank of a female, the higher the rank of her offspring [25]. For immature individuals with the same mother, the younger an individual is, the higher their social rank [25]. Previous studies have found that the percentage of individuals engaged in social play was 12.5% in the daily activity budgets during the immature periods [28], which increased during the infant period (peaked at approximately 12 months of age) and decreased during the juvenile period [28]. The rate of intrasexual grooming increased with age [28].

In this study, we divided the immatures into three age classes (i.e., infants, juveniles, and adolescents) to investigate the effects of the mother’s dominance hierarchy on the processes of participating in social interactions and choosing partnerships by comparing the social play and social grooming in Tibetan macaques. The following predictions were tested. If the mothers’ dominance hierarchy positively affects the social interactions of their immature offspring, we predicted that immatures with higher-ranking mothers would engage in more social play and grooming than those with lower-ranking mothers. If the mothers’ dominance hierarchy influences the partnership of their immatures, we predicted that the more similar the immatures’ mothers’ social ranks, the more social play they initiated with each other. However, the immatures with lower-ranking mothers were more likely to groom the immatures with higher-ranking mothers than vice versa.

## 2. Materials and Methods

### 2.1. Study Site and Subjects

This study was conducted at Mt. Huangshan, China. Several groups of Tibetan macaques are found throughout the reserve [29]. Beginning in 1987, we initiated observations of a single macaque study group (Yulinkeng A1, YA1). The study group was provisioned four times per day (a total of ca. 6 kg of dried corn per day) at the tourist viewing area during 2004 and 2014. Since 2014, the viewing site has been closed to tourists, and thus the subjects are wildlife.

This study was conducted from November 2020 to June 2021. During this period, the YA1 group was composed of 10 adult males (2 adult males immigrated, and 2 adult males emigrated), 14 adult females, 9 adolescents (1 adolescent died), 10 juveniles, and 14 infants (9 infants were born, but 1 died). The subject individuals included 9 adolescents (6 males and 3 females; males: 3–7 years old, females: 3–5 years old), 10 juveniles (6 males and 4 females; males/female: 1–3 years old), and 14 infants (9 males and 5 females; males/female: <1 year old) (see Table 1). More details about the subject monkeys can be found in Table 1. The age classes were derived from [25].

### 2.2. Data Collection

Using a digital video camera (model: SONY HDR-CX680) and a digital voice recorder (model: Lenovo B618), we collected behavioral data during November 2020 and June 2021, according to Xia et al. [30]. We collected all the data when the monkeys were in the natural forest to reduce the potential influence of human activity. We used the focal animal sampling method and continuous recording method to collect behavioral data of social play and social grooming [31]. Each focal sampling period was set as 10 min. When the focal monkey could not be followed or disappeared from view during the sampling period, we randomly selected another individual [18,32]. We recorded the behavior of the disappeared individual in the next 10 min of sampling time [33,34,35]. A total of 123 h of data (mean ± SE = 3.70 ± 0.21 h; n = 33; range: 1.00 ± 4.50 total h per monkey) was collected during the focal sampling period. In addition, we used an ad libitum approach to record aggressive and submissive interactions of adult females for determining dominant relationships.

### 2.3. Dominance Hierarchy

We calculated individual David’s scores for dominance hierarchies of adult females based on a dyadic aggressive/submission matrix, which showed the direction of agonistic interactions given and received. We used k-means cluster analysis (100 iterations) to classify each adult female into the following rank classes: high-ranking (YXX, YH, YCY, YXY), middle-ranking (YCH, TXH, YCL, TH), and low-ranking (TXX, TQL, HH, THY, THX, HXW) (see Table 1). In addition, we defined nine categories of mother–mother dyads based on rank classes (H-H: dyads consisting of two high-ranking mothers; H-M and M-H: dyads consisting of one high- and one middle-ranking mother; H-L and L-H: dyads consisting of one high- and one low-ranking mother; M-M: dyads consisting of two middle-ranking mothers; M-L and L-M: dyads consisting of one middle- and one low-ranking mother; and L-L: dyads consisting of two low-ranking mothers, with the former as initiator and the latter as receiver).

Aggressive behavior was defined as one individual threatening, chasing, slapping, grabbing, or biting another individual [36]. Submissive behavior was defined as an individual showing fearful behaviors, such as a fear grin, cower, mock leave, avoid, flee, or scream, as defined by Berman et al. [27].

### 2.4. Behavioral Definition

According to Burghardt’s identification criteria of social play, it was feasible to distinguish the difference between social play and non-play behaviors. Social play was defined as behaviors functioned to develop, practice, or maintain physical or cognitive abilities and social relationships, including tactics by varying, repeating, or recombining already functional sub-sequences of behavior outside their primary context [28]. We categorized them into aggressive play (rough pattern) and affiliative play (soft pattern) [28]. The aggressive play is usually in the form of individual slapping, wrestling, gently biting and chasing. The affiliative play is usually in the form of individual mounting, cuddling, and sucking a penis. The detailed definition of social play can be found in Wang et al. [28]. For data collection, a play episode began when two or more individuals came into direct contact and engaged in slapping or other play behaviors (see [28]). A play episode ended when all players started to initiate non-play activities (e.g., resting, social grooming, and travel) or withdrew from the episodes, or interference from adults resulted in all players stopping their play activities [37].

Social grooming was defined as any act in which an individual (groomer) used their hand or mouth to touch, clean, or manipulate the fur of another individual (groomee) for a continuous period lasting at least 5 s [30,38]. The detail of the recording rules can be found in Xia et al. [30,39].

### 2.5. Data Analysis

We report all data as the mean ± SE for the rate of social play (episodes/h), the percentage of aggressive and affiliative play, the rate of social grooming (bouts/h), the percentage of grooming initiated and grooming received, and the percentage of initiated play and grooming subject selections. They were calculated as individual behavioral data for each immature per month that a focal subject participated in social play or social grooming. We used a one-sample Kolmogorov–Smirnov test to examine the normality of the data (*p* > 0.05).

We used a Kruskal–Wallis H test to analyze differences in the rate of social play and social grooming engaged in by infant, juvenile, and adolescent offspring of high-, middle-, and low-ranking females. We also used a Kruskal–Wallis H test to determine differences in social play type and social grooming engaged in by infant, juvenile, and adolescent offspring of high-, middle-, and low-ranking females. We used a Mann–Whitney U test to compare them in pairs. In addition, we used a Kruskal–Wallis H test to determine the difference in initiated social play/grooming among infant, juvenile, and adolescent offspring with high-, middle-, and low-ranking mothers. We used a two-tailed test with the alpha set at 0.05. All the analyses were performed using the SPSS 26.0 software (SPSS Inc., Chicago, IL, USA).

## 3. Results

During the study period, 2156 episodes of social play among 33 immature individuals were recorded. Infants engaged in 944 (43.8%) play episodes, juveniles engaged in 1012 (46.9%) play episodes, and adolescents engaged in 200 (9.3%) play episodes. Among all play interactions, 1928 (89.4%) were aggressive play, and 228 (10.6%) were defined as affiliative play.

We also recorded 1166 bouts of social grooming among 33 immature individuals. Infants engaged in 169 (14.5%) grooming bouts, juveniles engaged in 470 (40.3%) grooming bouts, and adolescents engaged in 527 (46.2%) grooming bouts.

### 3.1. Effects of Mother’s Social Rank on Social Play among Immature Individuals

**Figure 1:**
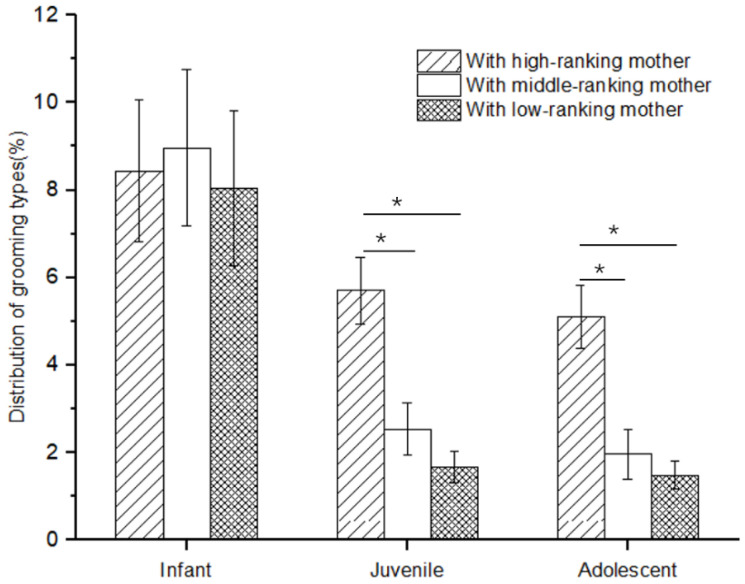
The rate of social play among immature individuals with mothers of different rank categories (* *p* < 0.05).

There was no difference in the rate of social play among infants with high-, middle-, and low-ranking mothers (Kruskal–Wallis H test, *χ*^2^ = 1.117, *p* = 0.572). However, there was a significant difference in the rate of social play among juveniles and adolescents with high-, middle-, and low-ranking mothers (juveniles: *χ*^2^ = 11.778, *p* = 0.03; adolescents: *χ*^2^ = 7.538, *p* = 0.023). Further analysis showed that the rate of social play of juveniles and adolescents with high-ranking mothers (juveniles: 5.71 ± 0.77; adolescents: 5.10 ± 0.72) was higher than that of juveniles and adolescents with middle-ranking mothers (juveniles: 2.54 ± 0.01; adolescents: 1.96 ± 0.01; Mann–Whitney U test, juveniles: *Z* = −2.458, *p* = 0.014; adolescents: *Z* = −2.309, *p* = 0.021) and low-ranking mothers (juveniles: 1.67 ± 0.37; adolescents: 1.48 ± 0.32; juveniles: *Z* = −3.37, *p* = 0.01; adolescents: *Z* = −2.309, *p* = 0.021), but not for the juveniles and adolescents with middle-ranking and low-ranking mothers (juveniles: *Z* = −0.34, *p* = 0.734; adolescents: *Z* = −0.577, *p* = 0.564; see Figure 1).

**Figure 2:**
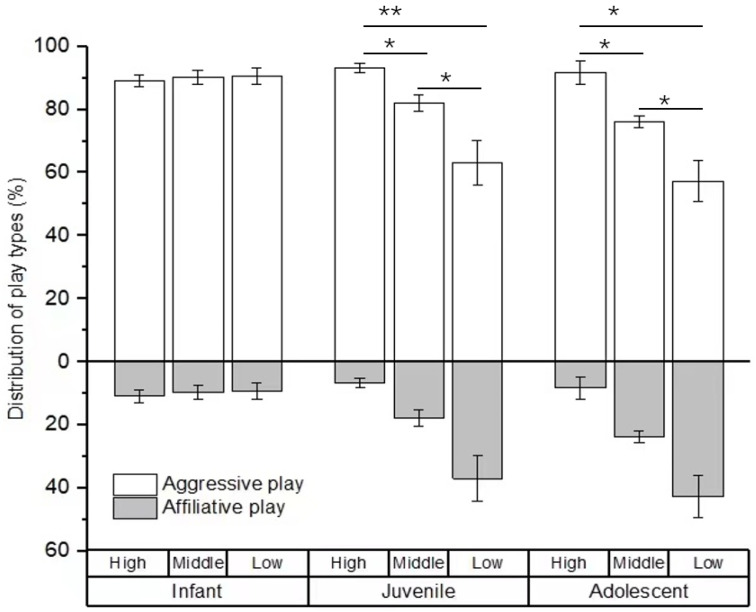
The percentage of play types among immature individuals with mothers of different rank categories. High, middle, and low refer to the social rank of immatures’ mothers (* *p* < 0.05; ** *p* < 0.01).

For the type of social play, no difference was found in the percentage of social play among infants with high-, middle-, and low-ranking mothers (*χ*^2^ = 0.594, *p* = 0.743). However, there were significant differences in the percentage of social play among juveniles and adolescents with high-, middle-, and low-ranking mothers (juveniles: *χ*^2^ = 19.108, *p* < 0.001; adolescents: *χ*^2^ = 7.538, *p* = 0.023). The juvenile and adolescent individuals with high-ranking mothers engaged in a higher rate of aggressive play (juveniles: 93.25 ± 1.57%; adolescents: 91.70 ± 3.58%) than those with middle-ranking mothers (juveniles: 82.14 ± 2.58%, *Z* = −2.956, *p* = 0.03; adolescents: 76.21 ± 1.90%, *Z* = −2.309, *p* = 0.021) and low-ranking mothers (juveniles: 63.05 ± 7.14%, *Z* = −3.784, *p* < 0.01; adolescents: 57.29 ± 10.54%, *Z* = −2.021, *p* = 0.043). We also found that the juvenile and adolescent individuals with middle-ranking mothers engaged in a higher rate of aggressive play than those with low-ranking mothers (juveniles: *Z* = −2.463, *p* = 0.014; adolescents: *Z* = −2.155, *p* = 0.022; see Figure 2).

**Figure 3:**
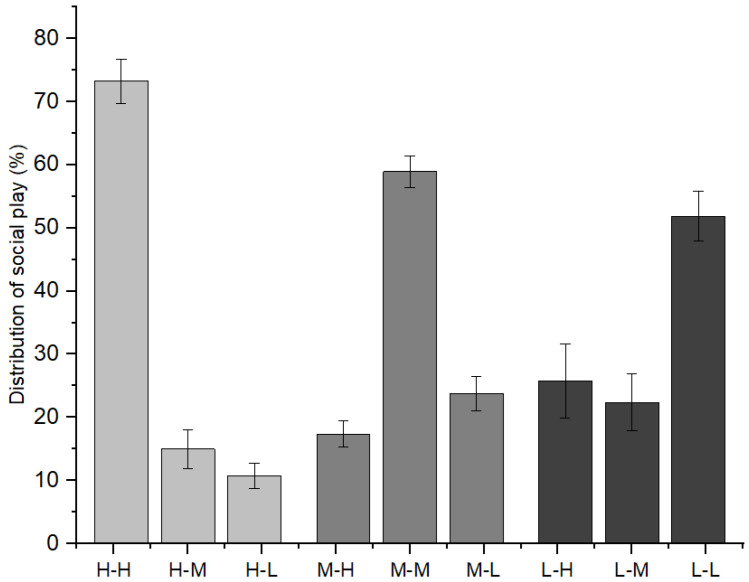
The relationships between playmates and the mothers’ social rank categories among immature individuals (H: high-ranking mother, M: middle-ranking mother, L: low-ranking mother).

Furthermore, as indicated in Figure 3, immature individuals with high-ranking mothers initiated 73.25 ± 3.53% of social play with immature individuals with high-ranking mothers, 15.00 ± 3.04% with those with middle-ranking mothers, and 11.76 ± 2.03% with those with low-ranking mothers (*χ*^2^ = 9.620, *p* = 0.008). Immature individuals with middle-ranking mothers initiated 17.37 ± 2.06% of social play with immature individuals with high-ranking mothers, 58.90 ± 2.48% with those with middle-ranking mothers, and 23.72 ± 2.71% with those with low-ranking mothers (*χ*^2^ = 7.639, *p* = 0.022). Immature individuals with low-ranking mothers initiated 25.74 ± 5.84% of social play with immature individuals with high-ranking mothers, 22.39 ± 4.55% with those with middle-ranking mothers, and 51.87 ± 3.94% with those with low-ranking mothers (χ^2^ = 6.478, *p* = 0.039).

### 3.2. Effects of Mother’s Social Rank on Social Grooming among Immature Individuals

**Figure 4:**
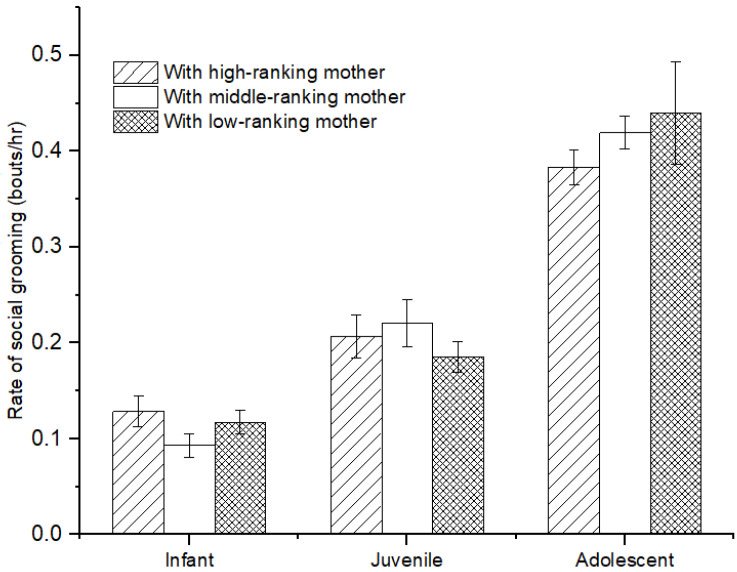
The rate of social grooming among immature individuals with mothers of different rank categories.

**Figure 5:**
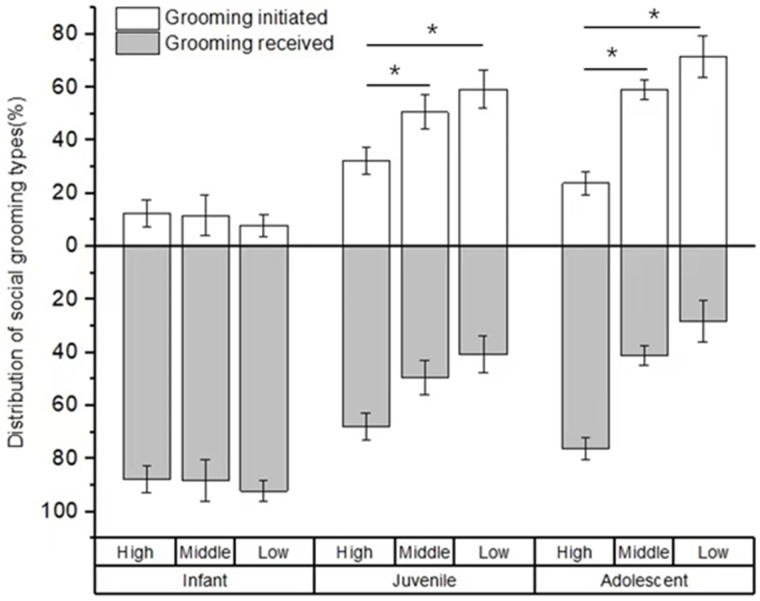
The percentage of grooming types among immature individuals with mothers of different rank categories. High, middle, and low refer to the social rank of immatures’ mothers (* *p* < 0.05).

There was no difference in the rate of social grooming among immatures with high-, middle-, and low-ranking mothers (infants: *χ*^2^ = 3.736, *p* = 0.154; juveniles: *χ*^2^ = 2.263, *p* = 0.323; adolescents: *χ*^2^ = 2.263, *p* = 0.323; see Figure 4). Furthermore, for infants, we also did not find a difference in social grooming engaged by immatures with high-, middle-, and low-ranking mothers (*χ*^2^ = 0.597, *p* = 0.742). However, there was a significant difference in social grooming initiated or received among juveniles and adolescents with high-, middle-, and low-ranking mothers (juveniles: *χ*^2^ = 7.206, *p* = 0.027; adolescents: *χ*^2^ = 8.000, *p* = 0.018). Juvenile and adolescent individuals with high-ranking mothers (juvenile: 67.83 ± 4.98%; adolescents: 76.21 ± 4.24%) initiated less or received more social grooming than those with middle-ranking mothers (juvenile: 49.44 ± 6.47%, *Z* = −2.09, *p* = 0.037; adolescents: 41.10 ± 3.70%, *Z* = −2.309, *p* = 0.029) and low-ranking mothers (juveniles: 40.75 ± 7.05%, *Z*= −2.496, *p* = 0.013; adolescents: 28.35 ± 7.84%, *Z* = −2.309, *p* = 0.029). No difference was found for social grooming initiated or received among juvenile and adolescent individuals with middle- and low-ranking mothers (juveniles: *Z* = −0.396, *p* = 0.713; adolescents: *Z* = −1.155, *p* = 0.343; see Figure 5).

**Figure 6:**
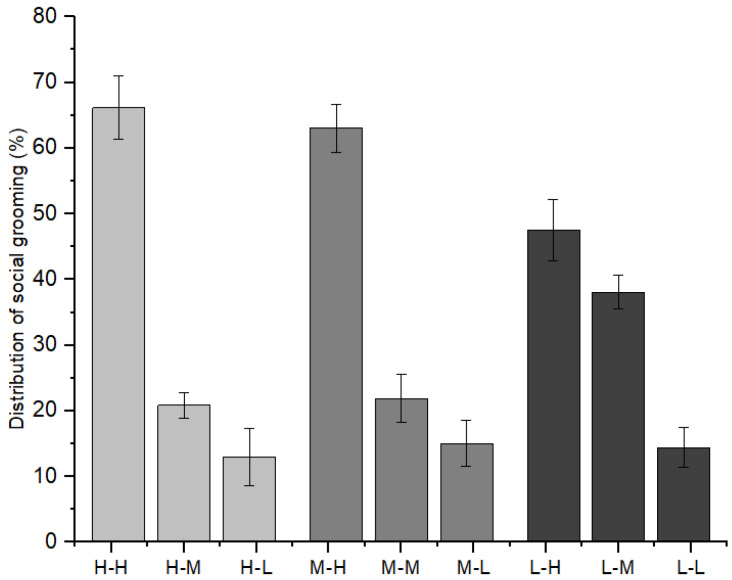
The relationships between grooming partners and the mothers’ social rank categories among immature individuals (H: high-ranking mother, M: middle-ranking mother, L: low-ranking mother).

Furthermore, as indicated in Figure 6, immature individuals with high-ranking mothers initiated 66.15 ± 4.80% of social grooming with immature individuals with high-ranking mothers, 20.89 ± 1.93% with those with middle-ranking mothers, and 12.96 ± 4.35% with those with low-ranking mothers (*χ*^2^ = 7.209, *p* = 0.027). Immature individuals with middle-ranking mothers initiated 63.01 ± 3.59% of social grooming with immature individuals with high-ranking mothers, 21.91 ± 3.61% with those with middle-ranking mothers, and 15.08 ± 3.52% with those with low-ranking mothers (*χ*^2^ = 8.782, *p* = 0.012). Immature individuals with low-ranking mothers initiated 47.54 ± 4.67% of social grooming with immature individuals with high-ranking mothers, 38.05 ± 2.58% with those with middle-ranking mothers, and 14.41 ± 3.01% with those with low-ranking mothers (*χ*^2^ = 8.103, *p* = 0.017).

## 4. Discussion

To our knowledge, this is the first study to investigate the effects of the mother’s dominance hierarchy on the processes of participating in social interactions and choosing partnerships by comparing the social play and grooming among infant, juvenile, and adolescent Tibetan macaques. We did not find a difference in social play and grooming between infants with high-, middle-, and low-ranking mothers. Juveniles and adolescents with high-ranking mothers engaged in more social play than those with lower-ranking mothers. The greater the similarity of their mothers’ social rank, the more social play they initiated with each other. Although there was no difference in grooming participated in by juveniles/adolescents with high-, middle-, and low-ranking mothers, juveniles and adolescents with high-ranking mothers initiated less or received more social grooming than those with middle- and low-ranking mothers. Furthermore, immatures preferred individuals with higher-ranking mothers as grooming mates. Our study suggests that the mother’s dominance hierarchy affects social play and grooming during the juvenile and adolescent periods, but not the infancy period. This indicates that the mother’s dominance hierarchy plays a varied role in the growth environment and the socialization processes among immature individuals in different growth stages (i.e., infancy, juvenile, and adolescent periods).

Our results show that, in the despotic Tibetan macaque society, the females’ dominance hierarchy has no effect on the social play and grooming of their infant offspring. However, a previous study found that high-ranking females are more laissez-faire towards their infants and low-ranking females are more protective of their infants in more despotic rhesus monkeys (*Macaca mulatta*) [40]. Thus, infants with high-ranking mothers engage in more social interaction, while infants with low-ranking mothers engage in less social interaction [20,40]. However, we have no data to show whether high-ranking or low-ranking mothers are rejective or protective in Tibetan macaques. Wright et al. reported that female Tibetan macaques rarely interrupted their infant offspring’s social interactions in the same group [41]. This result suggests that Tibetan macaques adopt a mothering style characterized by low restrictiveness contrary to the expectation for more despotic species (such as rhesus monkeys [40]). Therefore, the different ranks of mothers may show the same parenting style for their offspring during the infancy periods. Similar results were also found in other primate species. For example, in a study of wild golden snub-nosed monkeys (*Rhinopithecus roxellana*), Li et al. found that the mother’s social rank did not influence the social play among infants [21]. Accordingly, our results provide more evidence that the difference in the effect of the mother’s dominance hierarchy between species is related to the difference in the species-typical social structure and mother’s parenting style.

Our results imply that the juvenile and adolescent periods might be critical stages for immature individuals to learn or exercise social skills needed in adulthood. Juveniles and adolescents need to do more than survive for nutrition [4]. Thus, they need to prepare for better integration into the social group or for forming social relationships as adults [4]. As a result, they become more independent, more likely to explore the environment, and participate in social interactions (social play and social grooming) [20,40]. Meanwhile, the tolerance of group members would decrease when the immature individuals are in the juvenile and adolescent periods [18]. As such, juveniles and adolescents must learn and understand the social structure of the group. Additionally, the characteristics of each partner interact to adjust their fitness to better engage in social interaction and choose social partners to cope with the complex environment in future adulthood.

Our results highlight that the effects of the mother’s dominance hierarchy on immature individuals’ socialization appeared in Tibetan macaques when their offspring were in the juvenile and adolescent periods. Previous studies have proposed that the higher-ranking mothers can provide a better environment (such as protection and aggressive support) for their offspring to grow up in, making their offspring bolder and allowing them to engage in more social interactions [42]. In this study, we found that juveniles and adolescents with higher-ranking mothers engaged in a higher rate of social play than those with lower-ranking mothers. Moreover, juveniles and adolescents with higher-ranking mothers engaged in more aggressive play than those with lower-ranking mothers. A similar pattern was also found in studies of juvenile lowland gorillas (*Gorilla gorilla gorilla*) [43] and juvenile vervet monkeys (*Cereopitheeus aethiops*) [44]. This indicates that the mother’s dominance hierarchy affects the rate and the type of social play. Juvenile and adolescent Tibetan macaques of higher-ranking mothers would have more chances to participate in social play, and to establish and form social relationships in the future. They would be likely to participate in aggressive play to rehearse the ability to fight seriously later with conspecifics (the moto training hypothesis [45]). This social play pattern could be particularly fruitful in providing immediate or long-term feedback on the physical skills of developing individuals [46]. For example, for male immature Tibetan macaques, Wang et al. found that aggressive play facilitates the skills to integrate into a new social group when they mature [28]. Accordingly, our results suggest the mother’s dominance hierarchy might affect future integration for dispersed male immatures and strategies to obtain a higher social rank in a natal group for philopatric female immatures.

In addition, since social play and aggressive play could be effective for individuals, the coming question is how to choose partnership. Our results show that the greater the similarity of the mothers’ social rank among immature dyads, the more social play they initiated with each other. This suggests that social play is helpful for immatures to choose a well-matched partner according to their mothers’ dominance hierarchy. In order to play without incurring severe aggression or injury, immatures must know all the characteristics of each immature individual and the social ranks of their mothers. This tendency is evident in many primate species (baboons, *Papio cynocephalus* [47,48]; rhesus macaques, *Macaca* *mulatta* [49]; chimpanzees, *Pan troglodytes* [50,51]; gorillas, *Gorilla gorilla gorilla* [52]). Previous studies proposed that symmetry characterizing play sessions among matched partners allows playmates to compete, practice, and strategize in a safer context [53]. Immatures of higher-ranking mothers grow faster and have greater physical strength, whereas immatures of lower-ranking mothers grow slower and have lower physical strength [24]. Accordingly, two playmates whose mothers are of a similar social rank might be fit enough to play together in order to test and improve their strength and motor ability due to the symmetry characterizing the two playmates [54].

Among juvenile and adolescent dyads, the present study also found that the effects of the dominance hierarchy on grooming patterns were similar to those among adult dyads. In nonhuman primates, previous studies demonstrated that lower-ranking adults initiated more social grooming with higher-ranking adults than vice versa (e.g., mandrills, *Mandrillus sphinx* [55]; Tibetan macaques, *Macaca thibetana* [30,39]). Social grooming is used to establish and maintain social relationships among immature individuals, which provides a better basis for group identification in adulthood [56]. However, less is known about the patterns of similarity between immature individuals and adults through social transmission whereby offspring learn the behavior from observing their mother or others [57,58]. Our results suggest that patterns of affiliative interactions (social grooming) between individuals begin to emerge in the juvenile and adolescent periods, not in adulthood.

## 5. Conclusions

The dominance hierarchy of adult females influences the rate of social interaction, the type or the number of social behaviors, and the social interaction partnerships among immature individuals. The present study provides new insight into understanding the effects of the mother’s dominance hierarchy on the development of social relationships among immature nonhuman primates. Future studies would be necessary to further explore the influence of maternal styles (i.e., protectiveness or rejection), the sex-specific strategies of male or female offspring, and the long-term effects of the female dominance hierarchy on the social strategies of immature individuals (i.e., mother’s social rank, social relationships, and reproductive success in the future).

## Figures and Tables

**Table 1:** Immature individuals in group YA1 during the study period.

Immature ID	Sex	Date of Birth	Age Class	Mother’s ID	Mother’s Rank Class	Mother’s NDS
YQT	Male	7 January 2018	Adolescent	YXX	High	81.00
YXP	Male	21 May 2018	Adolescent	YCY	High	56.67
YQX	Male	4 April 2019	Juvenile	YXX	High	81.00
YXQ	Female	9 May 2019	Juvenile	YH	High	67.67
YQS	Male	30 June 2019	Juvenile	YXY	High	40.75
YXZ	Male	?? March 2020	Infant	YCY	High	56.67
YQC	Male	?? April 2020	Infant	YXX	High	81.00
YQQ	Female	12 March 2021	Infant	YXY	High	40.75
YXC	Male	20 March 2021	Infant	YCY	High	56.67
TQS	Male	11 April 2015	Adolescent	TXH	Middle	9.90
YXM	Male	11 March 2017	Adolescent	YCL	Middle	8.17
TQG	Female	18 June 2017	Adolescent	TXH	Middle	9.90
TXJ	Male	1 August 2017	Adolescent	TH	Middle	−10.00
TXD	Female	27 February 2019	Juvenile	TH	Middle	−10.00
TQZ	Female	3 March 2019	Juvenile	TXH	Middle	9.90
TXC	Male	?? April 2020	Infant	TH	Middle	−10.00
TQT	Male	23 February 2021	Infant	TXH	Middle	9.90
YXT	Female	30 March 2021	Infant	YCL	Middle	8.17
YXL	Female	30 March 2021	Infant	YCH	Middle	30.00
TQY	Female	4 March 2016	Adolescent	TXX	Low	−23.77
TFH	Female	27 March 2016	Adolescent	THY	Low	−45.27
TFK	Male	13 February 2018	Adolescent	THY	Low	−45.27
TFJ	Male	25 March 2018	Juvenile	THX	Low	−63.58
HXC	Male	12 June 2018	Juvenile	HH	Low	−44.43
TQM	Male	24 February 2019	Juvenile	TXX	Low	−23.77
TFZ	Male	2 May 2019	Juvenile	THY	Low	−45.27
HQZ	Female	11 February 2019	Juvenile	HXW	Low	−80.67
TFC	Male	?? April 2020	Infant	THX	Low	−63.58
TFY	Male	29 May 2020	Infant	THY	Low	−45.27
HQG	Male	2 March 2021	Infant	HXW	Low	−80.67
HQX	Male	1 April 2021	Infant	HXY	Low	−45.46
HXS	Female	13 March 2021	Infant	HH	Low	−44.53
TQW	Female	2 April 2021	Infant	TXX	Low	−23.77

Note: NDS: normalized David’s score (see below Section 2.3).

## Data Availability

The data that support the findings of this study are available on request from the corresponding author; the data are not publicly available due to privacy or ethical restrictions.
